# Association of serum thyrotropin levels with coronary artery disease documented by quantitative coronary angiography: a transversal study

**DOI:** 10.20945/2359-3997000000054

**Published:** 2018-08-01

**Authors:** Pedro D. Ortolani, João H. Romaldini, Ricardo A. Guerra, Evandro S. Portes, George C. X. Meireles, João Pimenta

**Affiliations:** 1 Instituto de Assistência Médica ao Servidor Público Estadual Instituto de Assistência Médica ao Servidor Público Estadual Hospital do Servidor Público Estadual de São Paulo Serviço de Endocrinologia São Paulo SP Brasil Serviço de Endocrinologia, Hospital do Servidor Público Estadual de São Paulo, Instituto de Assistência Médica ao Servidor Público Estadual (HSPE-IAMSPE), São Paulo, SP, Brasil; 2 Instituto de Assistência Médica ao Servidor Público Estadual Instituto de Assistência Médica ao Servidor Público Estadual Hospital do Servidor Público Estadual de São Paulo Serviço de Cardiologia São Paulo SP Brasil Serviço de Cardiologia, Hospital do Servidor Público Estadual de São Paulo, Instituto de Assistência Médica ao Servidor Público Estadual (HSPE-IAMSPE), São Paulo, SP, Brasil

**Keywords:** TSH, coronary artery disease, coronary angiography

## Abstract

**Objective:**

The association between coronary artery disease (CAD) and thyroid function remains controversial. We evaluated the thyroid function and graduated well-defined CAD as confirmed by quantitative coronary angiography (CA).

**Subjects and methods:**

We evaluated the serum TSH, free thyroxine, free triiodothyronine and thyroid antibody levels in 300 consecutive patients (age 61.6 ± 9.9 years and 54% were male) undergoing CAD diagnosis as confirmed by CA. Plaques with ≥ 50% stenosis being indicative of obstructive CAD, and patients were divided into groups according to main epicardial coronary arteries with plaques (0, 1, 2, 3). Lipid profiles and a homeostasis model assessment (HOMA-IR) were determined.

**Results:**

Serum median (25% and 75% percentile) TSH levels in patients with group 2 and 3 (2.25; 1.66-3.12 mU/L and 4.99; 4.38-23.60 mU/L, respectively) had significantly higher TSH concentrations (p < 0.0001) than the group 0 (1.82; 1.35-2.51 mU/L). Furthermore, patients of group 3 had higher TSH concentration (p < 0.0001) than those of group 1 (1.60; 0.89-2.68 mU/L). Group 3 were older (64 ± 8.5 vs. 59 ± 9.5, p = 0.001), had more patients with dyslipidemia (84% versus 58%, p < 0.001), male (54% versus 44%, p = 0.01), hypertension (100% versus 86%, p < 0.001), and smoking (61% versus 33%, p < 0.001) than group 0. Multivariate stepwise logistic analysis showed TSH, age, HbA1c, and HOMA-IR were the CAD associated variables.

**Conclusions:**

In this cohort, elevated TSH levels in the high normal range or above are associated with the presence and severity of CAD besides may represent a weak CAD risk factor.

## INTRODUCTION

Hypothyroidism is typically associated with elevated endothelial dysfunction and increased all-cause mortality in addition to cardiac death and/or hospitalization (1-3). Several studies have shown that coronary atherosclerosis in subjects with hypothyroidism is more frequent and severe than in normal subjects (4). In addition, there is some evidence that an elevated serum TSH in association with serum thyroid peroxidase antibody (TPOAb) may alter the traditional risk factors for coronary arterial disease (CAD), perhaps because of the accumulation of circulating levels of atherogenic total cholesterol (TC) and low-density lipoprotein (LDL) cholesterol particles (5-9). Consequently, the relation between thyroid dysfunction and CAD remains questionable (10), however, some authors have found a positive correlation between serum TSH levels and the presence and severity of coronary stenosis (6,11-14); other authors have found no correlation between these two parameters (15,16). In contrast, Coceani and cols. observed a negative correlation with serum FT3 (but not with FT4 levels) and CAD (17). On the other hand, Miura and cols. found that independent of conventional cardiovascular risk factors, thyroid hormones (FT3 and FT4) inversely correlated with coronary artery disease, especially men (18,19). A meta-analysis demonstrated that high TSH values were associated with cardiovascular risk in patients < 65 years old, whereas another study reported that in patients > 85 years old, increased serum TSH values played a protective role against the development of CAD (20,21). Furthermore, it remains to be determined whether serum TSH, FT4, FT3, TPOAb or thyroglobulin antibody (TgAb) levels are associated with CAD development. The aim of this study was to yield greater insight into the assessment of thyroid function parameters and lipid concentrations in consecutive patients undergoing quantitative coronary angiography (CA).

## SUBJECTS AND METHODS

### Patients

Data were obtained from 343 consecutive patients in the Cardiology Outpatient Unit, *Hospital do Servidor Público Estadual de São Paulo, Instituto de Assistência Médica ao Servidor Público Estadual* (HSPE-IAMSPE) in Brazil that were referred for CA because of signs or symptoms relevant to CAD. All of the patients satisfied two inclusion criteria: 1) characteristic signs of myocardial ischemia on non-invasive tests and 2) clinical recommendation for further assessment. We used a questionnaire to assess classical risk factors for CAD such as diabetes, hypertension, dyslipidemia, smoking, and a family history of premature CAD (defined as ≤ 55 years old for males and ≤ 65 years old for females). Patients were excluded in cases with a history of thyroid diseases, documented CAD, acute CAD, or ischemic equivalent (such as dyspnea, sickness, confusion, stroke or pulmonary edema, compatible electrocardiographic signs, or increased creatine kinase-MB [CK-MB)] and/or troponin levels) (22). We excluded patients who were using amiodarone, levothyroxine, or antithyroid drugs (such as methimazole or propylthiouracil). Forty-three patients were excluded, and the remaining 300 patients were enrolled in the study. All of the patients included in the study received an explanation of the purpose of the study and signed a consent form. This work was submitted to the Research Ethics Committee of IAMSPE and approved without restrictions.

### Assessment of CA

All of the patients were assessed via CA and further analyzed by an independent observer who was not involved in patients medical care. The quantification of the CA was performed manually. The CA was carried out using a femoral approach and a 6- or 7-F guiding catheter. Right and left CAs were performed in multiple projections using standard techniques and used to evaluate coronary artery stenosis (23). CAD was defined according to CA results, and significant stenosis was defined as a decrease in internal diameter of ≥ 50%, which could affect one or more of the main epicardial coronary arteries (such as the right coronary, left anterior descending, or circumflex arteries) and classified as group 1, 2, or 3 according the number of vessels with significant stenosis (24). Patients were classified as group 0 if they had no CAD or when the main epicardial coronary arteries were < 50% of the internal diameter.

### Biochemical analysis

Blood samples were drawn after overnight fasting prior to the CA. For the hormone assessment, patient samples were stored at −20°C until the time of measurement and analyzed in our laboratory. Blood glucose levels were estimated using the glucose oxidase method. Hemoglobin A1c (HbA1c), CK-MB, and cardiac troponin levels were determined by HPLC, electrochemiluminescent immunoassay, and two-site enzyme-linked immunosorbent assay methods, respectively. Serum TC, triglycerides (TG), and high-density lipoprotein (HDL) cholesterol levels were measured enzymatically. To determine LDL-cholesterol levels, the formula from Friedewald and cols. was used (25). Serum creatinine level was analyzed using a colorimetric method, and hepatic enzyme levels were determined enzymatically.

### Hormonal analysis

Serum TSH, FT4, and FT3 levels were measured using direct chemiluminescent technology (Advia Centaur XP Immunoassay System; Siemens Healthcare Diagnostics, Deerfield, IL, USA) using reference values ranging from 0.3-4.1 mU/L for TSH, 0.8-2.0 ng/dL for FT4, and 1.6-4.1 pg/mL for FT3. The mean interassay CV was 3.1, 11.0, and 8.9 % for TSH, FT4, and FT3, respectively. TPOAb and TgAb serum concentrations were determined via a chemiluminescent immunometric assay (Diagnostic Products Co., Los Angeles, CA, USA), and values > 35 IU/L were considered to be positive. Serum insulin was analyzed using a chemiluminescent immunometric assay (Diagnostic Products Co.). The homeostasis model assessment of insulin resistance (HOMA-IR) index was determined using serum glucose and insulin values: fasting insulin (mU/mL)×fasting blood glucose (mg/dL)/405. Body mass index (BMI) was expressed as kg/m^2^.

### Statistical analysis

Differences between the groups were verified by the Kruskal-Wallis in conjunction with the Dunn's multiple comparison tests, and thereafter they were compared two-by-two using the Mann-Whitney rank test. Chi-square or Fischer's exact tests were used to compare proportions Univariate and multivariate analyses with multinomial logistic regression analysis were used to evaluate the risks associated with CA results. For univariate analysis a less-restrictive alpha level with a 0.1 was used, and so a broad range of variables are considered for inclusion in the model. Thereafter, a backward stepwise multiple regression analysis was employed using CAD as dependent variable. A beta coefficient, standard error of beta coefficient (SE), and 95% confidence interval were obtained. SAS software version 9.4 (SAS Institute Inc., Cary, NC) was used for all analyses.

## RESULTS

**Figure 1:**
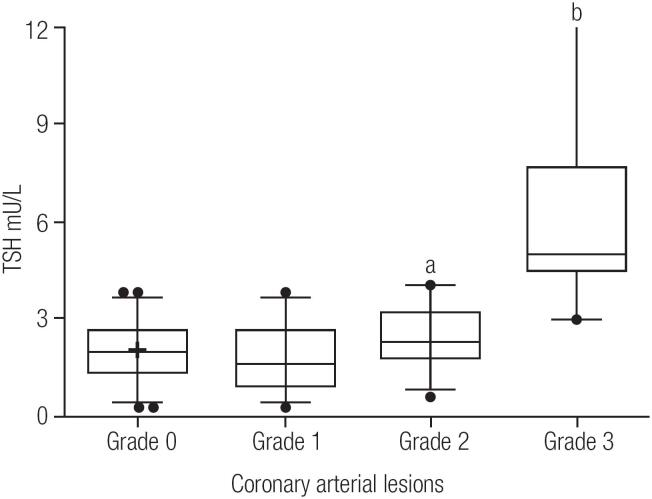
Box- and-whisker plot of the differences in the serum TSH median observed between patients undergoing the coronary angiography. Group 0; absence of coronary artery disease (when the vessels were < 50% of the internal diameter). Group 1, 2, and 3; according to the number of vessels with significant stenosis. The median is the center line, the ends of the box represent the 25^th^ and 75^th^ percentiles, and the ends of the lines extend to the 2.5^th^ and 97.5^th^ percentiles. Differences between the groups were analyzed by the Kruskal-Wallis test. (H = 203.5; p < 0.0001). ^a^ p < 0.02 vs. group 0; ^b^ p < 0.0001 vs. group 0, 1 and 2 (Verified by the Mann-Whitney test was used).

Characteristics of patients that were analyzed and patient demographics are reported in Table 1. All enrolled patients underwent all tests according to schedule. The mean age was of 61.6 ± 9.9, > 90% of patients had hypertension, 72% had dyslipidemia, and almost half of them were smokers. Mean serum TSH levels were 3.0 ± 2.7 (SD) mU/L, and 11% of patients had positive thyroid antibodies. Significant stenosis (≥ 50% in one or more main epicardial vessels) was observed in 62% (185) of patients. Arterial vessel stenosis was observed in 60 patients (group 1), 64 patients had two stenosed vessels (group 2), and 61 patients had three stenosed vessels (group 3). Figure 1 shows the results of serum TSH concentration in patients undergoing CA examination. Serum median and a 25% and 75% percentile of TSH levels in patients of group 2 (2.25; 1.66-3.12 mU/L) and group 3 (4.99; 4.38-23.60 mU/L) had significantly higher TSH concentrations (p < 0.0001) than the group 0 (1.82; 1.35-2.51 mU/L). Furthermore, patients of group 3 had higher TSH concentration (p < 0.0001) than those of group1 (1.60; 0.89-2.68 mU/L) as shown Figure 1. Moreover, group 3 had significant more patients with dyslipidemia, hypertension and smoking than group 0 patients. In group 3, the mean HbA1c levels were higher than other groups, and were older as shown Table 2. The group 2 had similar figure; had more male, smoking and diabetes patients as well as high HbA1c level, and older than group 0. Univariate logistical regression analysis revealed that male sex, diabetes mellitus, hypertension, dyslipidemia, smoking, older age and TSH were significant risk factors for CAD (Table 3). Thereafter, as shown in Table 4, the stepwise backward multiple regression model of CAD identified TSH, older age, HbA1c, and HOMA-IR as CAD risk factors (R^2^ = 0.377; Adjusted R^2^ = 0.369; SE = 0.922 F = 42.526; p-value = 0.0001).

**Table 1:** Baseline characteristics of 300 patients participants in the study

Variables	Number (%) or mean ± SD
Age, years	61.6 ± 9.9
Male	162 (54)
Diabetes mellitus	107 (36)
Hypertension	275 (92)
Smoking	147 (49)
Family history of CAD^a^	70 (23)
Dyslipidemia	215 (72)
BMI, kg/m^2^	28.2 ± 4.5
Total cholesterolemia, mg/dL	178.7 ± 37.9
HDL-cholesterolemia, mg/dL	49.1 ± 14.1
Trygliceridemia, mg/dL	134 ± 73.1
HbA1c, %	6.3 ± 1.4
LDL-cholesterolemia, mg/dL	101 ± 31.9
Thyroid antibodies^b^	34 (11)
TSH, mU/L	3.0 ± 2.7
FT4, ng/dL	1.2 ± 0.4
FT3, ng/dL	2.9 ± 0.6

SD: standard deviation.

a CAD coronary artery disease.

b Presence of serum thyroglobulin antibody and thyroid peroxidase antibody.

**Table 2:** Characteristics of patients according to the coronary artery angiography

Variables	Group 0^a^	Group 1	Group 2	p^b^	Group 3	p^c^
number	115	60	64		61	
Age, mean ± SD	59 ± 9.5	61.4 ± 10.1	63.8 ± 10.6	0.002	64.7 ± 8.5	0.001
Male, n (%)	51 (44)	31 (52)	36 (56)	0.015	33 (54)	0.01
Diabetes mellitus, n (%)	31 (27)	19 (32)	25 (39)	0.0001	33 (54)	0.107
Hypertension, n (%)	99 (86)	55 (92)	60 (94)	0.142	61 (100)	0.0001
Smoking, n (%)	38 (33)	32 (53)	39 (65)	0.001	37 (61)	0.0007
Familiar history of CAD	24 (21)	17 (28)	13 (20)	1.001	17 (28)	0.259
Dyslipidemia, n (%)	67 (58)	44 (73)	54 (84)	0.142	51 (84)	0.0001
BMI, Kg/m^2^, mean ± SD	29.0 ± 4.9	27.2 ± 4.0	27.5 ± 4.3	0.059	28.3 ± 3.9	0.097
Total cholesterolemia, mg/dL	185.2 ± 35.2	174.7 ± 39.1	174.4 ± 39.2	0.610	175.3 ± 38.9	0.088
HDL-cholesterolemia, mg/dL	50.3 ± 11.7	49.7 ± 12.4	48.0 ± 15.7	0.210	47.4 ± 12.7	0.130
Trygliceridemia, mg/dL	131 ± 70.6	136.3 ± 83.2	133.3 ± 65.4	0.450	137.6 ± 76.2	0.213
LDL-cholesterolemia, mg/dL	108 ± 32.5	97.7 ± 33.7	99.5 ± 29.8	0.750	100.5 ± 33.1	0.923
HbA1c, %, mean ± SD	6.0 ± 1.2	6.1 ± 1.3	6.5 ± 1.4	0.002	6.7 ± 1.8	0.018
Thyroid antibodies^d^, n (%)	12 (10)	9 (15)	7 (10.9)	0.607	7 (11.4)	0.596
TSH, mU/L	2.7 ± 2.1	3.1 ± 2.6	2.9 ± 2.1	0.025	3.5 ± 3.8	0.0001
FT4, ng/dL	1.1 ± 0.2	1.1 ± 0.2	1.2 ± 0.2	0.139	1.1 ± 0.2	0.758
FT3, ng/dL	2.9 ± 0.5	2.9 ± 0.6	3.0 ± 0.5	0.254	2.9 ± 0.5	0.800

SD: standard deviation.

a Group was classified as the number of main epicardial vessels with stenosis: Group 0 when vessels without lesion or less than 50% of the internal diameter, Group 1 when one vessel with lesion equal or more than 50% of the internal diameter, Group 2 when two vessels with lesion equal or more than 50% of the internal diameter, Group 3 when three vessels with lesion equal or more than 50% of the internal diameter.

b p-values significant between patients with Group 0 and Group-2, and no significant with Group 1.

c p-values significant between patients with Group 0 and Group 3, and no significant with Group 1 and Group 2.

d Presence of serum thyroglobulin antibody and thyroid peroxidase antibody Kruskal-Wallis was used (H = 7357.8; p < 0.00010. Mann-Whitney test was used for continuous variables between the groups, and the Chi-Square or Fischer's exact test was used for categorical variables.

**Table 3:** Risk factors associated with coronary arterial disease in patients undergoing coronary angiography by univariate logistic regression analysis

Variable	Adjusted odds ratio	95% Confidence Interval	p
Age, years	1.05	1.02-1.07	< 0.01
Male	1.93	1.27-2.93	< 0.01
Diabetes mellitus	1.17	1.41-3.34	< 0.01
Hypertension	3.55	1.53-8.24	< 0.01
Dyslipidemia	2.74	1.70-4.42	< 0.01
Smoking	2.35	1.54-3.57	< 0.01
HOMA-IR	1.12	1.05-1.20	< 0.01
HbA1c	1.32	1.13-1.54	< 0.01
TSH	1.07	0.99-1.16	= 0.08

Variables such as familiar history of CAD (p = 0.45), BMI (p = 0.21), thyroid antibodies (p = 0.78), FT4 (p = 0.58) and FT3 were not independent risk associated with CAD.

**Table 4:** Result from stepwise backward regression analysis for presence of coronary arterial disease in patients undergoing coronary angiography

Variables	Coefficients	SE^a^	Beta	*t*	p
TSH	0.238544459	0.021348719	0.52981	11.173	0.0008
Age	0.018485266	0.005520596	0.159255363	3.348	0.0009
HbA1c^b^	0.454384746	0.121113561	0.17814673	3.752	0.0002
HOMA-IR^c^	0,026698419	0.11200489	0.112782451	2.38	0.017

a SE, the estimated standard deviation of the error in the model.

b Hemoglobin A1c.

c The homeostasis model assessment of insulin resistance.

Independent variables such as sex (p = 0.31), diabetes mellitus (p = 0.27), hypertension (p = 0.33), dyslipidemia (0.25) and smoking (p = 0.94) were not included in the model.

## DISCUSSION

In this study, we have demonstrated that within our study population, patients with two or three stenosed arterial vessels at the CA assessment had higher serum TSH levels than patients with no stenosis. This study shown that serum TSH level was associated with risk of stenosis, detected during the CA, especially for patients with older age, higher levels of HbA1c and presence of insulin resistance. Serum FT4 or FT3 levels had no effect on the presence of stenosis. Recent meta-analysis provided some important information about the role of thyroid autoimmunity as a possible risk factor for CAD and suggested that biomarkers of thyroid autoimmunity do not add independent prognostic information for CAD outcomes (26). In the present study population, there was no association between significant stenosis seen on the CA and the presence of circulating TPOAb or TgAb. Although our findings using stepwise backward multiple regression model of CAD identified TSH, older age, HbA1c, and HOMA-IR as CAD predictors besides TSH were a weak independent risk factor for CAD. These data are consistent with the hypothesis that serum TSH values, even within in the upper normal reference range, are associated with the presence of stenosis (10). Endothelial dysfunction has been detected in hypothyroid patients, and it has been shown to be associated with low-intensity chronic inflammation, arterial vasodilatation reduction, and the presence of circulating TPOAb or TgAb (27,28). A limitation of our study was the small number of enrolled patients, which reduced the strength of the reported associations. Furthermore, in an attempt to decrease the influence of acute coronary syndrome, we included only select patients without clinical symptoms of cardiac disease such as chest pain or patients with manifestations of ischemic heart disease. On the other hand, it seems that there was no clear agreement on the association of serum TSH, FT4, and FT3 values with CAD. A multicenter prospective study, including 55,000 patients, revealed that elevated serum TSH levels (particularly levels > 10 mU/L) were associated with an increased risk of cardiac events and mortality due to CAD, and Chaker and cols. found that high levels of TSH might increase the risk of stroke in patients < 65 years old (29,30). Auer and cols. identified a positive correlation between serum TSH levels and the presence and severity of CAD without excluding patients with previous thyroid disease (31). Miura and cols. studied angina patients found in CAD patients lower FT4 and FT3 levels as compared to patients without coronary stenosis (18). On the contrary, Coceani and cols. assessed approximately 1,000 patients (excluding individuals with acute myocardial infarction) and found no correlation between serum TSH or FT4 levels and CAD and observed a negative correlation only with serum FT3 levels (17). We did not find any association between serum FT4 and/or FT3 levels and CAD. A direct correlation between TC and LDL cholesterol is well-known, even when serum TSH values are within the reference range, and is usually associated with insulin resistance and in smokers (8,32). Among the many factors associated with CAD, Tatar and cols. reported a direct effect of serum TSH in the upper normal reference range on an increase in arterial stiffness in dialysis patients with euthyroidism (33). On the other hand, serum TSH also increased inflammatory molecules and inhibited nitric oxide production due to oxidative stress as demonstrated by Desideri and cols. and Dardano and cols. (34,35). We were unable to find any evidence for an association between lipid profiles and serum TSH levels, most likely due to the fact that the majority of patients were taking dyslipidemia drugs. Despite the elevated frequency of hypertensive patients in our study population, no association with serum TSH values was noted.

Some paper found results similar to ours study: Yun and cols. used a serum TSH cutoff point of 2.1 mU/L; observed a significant correlation between serum TSH levels and coronary artery lesions in patients with unstable angina (36). Furthermore, using the median serum TSH value (1.73 mU/L) as a cutoff value, Yang and cols. studied the hospital-based diagnoses of myocardial infarction in 318 patients and found an association between elevated serum TSH values (still within the reference range) and CAD (37). Daswani and cols. found lower FT3 levels in patients with three stenosed vessels were more affected in CAD than in normal patients (38). Finally, the possibility that serum TSH levels is a cardiovascular risk factor has been a subject of debate, and a broad studies are needed for answer this point. In summary, our study suggests that in a population with multiple risk factors for CAD development, there is an association with high serum TSH, even in the upper normal range, with presence and severity of CAD as detected with CA. Furthermore, elevated serum TSH may represent a weak CAD risk factor.
